# Emerging concepts on Leydig cell development in fetal and adult testis

**DOI:** 10.3389/fendo.2022.1086276

**Published:** 2023-01-04

**Authors:** Indrashis Bhattacharya, Souvik Dey

**Affiliations:** ^1^Department of Zoology, School of Biological Science, Central University of Kerala, Periye, Kerala, India; ^2^Manipal Centre for Biotherapeutics Research, Manipal Academy of Higher Education, Manipal, Karnataka, India

**Keywords:** testosterone, fetal Leydig cell, adult Leydig cell, neonatal testis, progenitor

## Abstract

Leydig cells (Lc) reside in the interstitial compartment of the testis and are the target of Luteinising hormone (LH) for Testosterone (T) production, thus critically regulates male fertility. Classical histological studies have identified two morphologically different populations of Lc during testicular development [fetal (FLc) and adult (ALc)]. Recent progress in *ex vivo* cell/organ culture, genome-wide analysis, genetically manipulated mouse models, lineage tracing, and single-cell RNA-seq experiments have revealed the diverse cellular origins with differential transcriptomic and distinct steroidogenic outputs of these populations. FLc originates from both coelomic epithelium and notch-active Nestin-positive perivascular cells located at the gonad–mesonephros borders, and get specified as Nr5a1 (previously known as Ad4BP/SF-1) expressing cells by embryonic age (E) 12.5 days in fetal mouse testes. These cells produce androstenedione (precursor of T, due to lack of HSD17β3 enzyme) and play critical a role in initial virilization and patterning of the male external genitalia. However, in neonatal testis, FLc undergoes massive regression/dedifferentiation and gradually gets replaced by T-producing ALc. Very recent studies suggest a small fraction (5-20%) of FLc still persists in adult testis. Both Nestin-positive perivascular cells and FLc are considered to be the progenitor populations for ALc. This minireview article summarizes the current understanding of Lc development in fetal and adult testes highlighting their common or diverse cellular (progenitor/stem) origins with respective functional significance in both rodents and primates. (227 words)

## 1 Introduction

Testosterone (T), which is produced by testicular Leydig cells (Lc), is essential for the fetal differentiation of male reproductive track, virilization of male external genitalia, pubertal maturation of testicular Sertoli cells (Sc) followed by meiotic progression of male germ cells (Gc) and spermiation, and controls sex drive/libido, making it an absolutely indispensable factor for male fertility (1). Historically, in 1850, German anatomist Franz von Leydig identified these cells in the interstitial compartment of seminiferous tubules (2). Bouin and Ancel suggested that androgens are produced by the interstitial Lc in 1903 (3). During the 1960s, Hall & Eik-Nes (4) and Ewing & Eik-Nes (5) independently proposed the involvement of pituitary gonadotropins in synthesis of testicular androgens. Finally, in 1969, Hall et al., demonstrated the bioconversion of T from cholesterol by these cells (6).

Classical histological studies have identified two morphologically distinct sub-populations of Lc during testicular development (7–10). In fetal mouse testis, Lc gets specified soon after sex determination (8–10). The expansion of fetal Leydig cells (FLc) occurs throughout the *in utero* life, peaking during birth, gradually declines and subsequently disappears during neonatal/pre-pubertal life (8–10). During pubertal testicular maturation, a second population known as Adult Leydig cells (ALc) get differentiated and colonize the testicular interstitium to support masculinity and male fertility throughout adulthood (8–10). The ALc are stimulated by luteinizing hormone (LH); LH binds its cognate receptor (LH-R, a typical G protein coupled receptor) on the ALc and initiate cAMP signaling, which in turn activates the protein kinase A (PKA) to induce expression of steroidogenic acute regulatory protein (STAR). STAR operates on mitochondria to stimulate cholesterol transport from outer to inner membranes and thereby initiates the bioconversion of pregnenolone by CYP11A and subsequently leading to synthesis of cholesterol. However, both of the Lc populations are significantly different in terms of origin, morphology, histology, and physiology (8–10). This article critically discusses the fundamental concepts of Lc differentiation during fetal and post-natal testicular development.

## 2 FLc

### 2.1 Origin

In mice, the nascent bipotential gonads arise from the coelomic epithelial layers surrounding the mesonephros, at embryonic age (E) E 9.5-10.5 days (10, 11). The transcriptional cascade of sex determination gets triggered with activation of *Sry* gene by Sc in XY embryos at E 11.5 days (11). FLc get specified in XY gonads by E 12.5 days (11). Multiple cellular origins have been demonstrated in contributing to the FLc population e.g.- coelomic epithelium, neural crest, notch active, Nestin-positive perivascular cells located at the gonad-mesonephros border region (12, 13) or interstitial nuclear receptor subfamily 5, group A, member 1 (Nr5a1or Ad4BP/SF-1)-positive (14) and WT1 (Wilms’ Tumor Gene 1) - positive stem/progenitor cells (15) etc.

### 2.2 Differentiation

The FLc expands in number throughout the fetal life, peaking around birth (10). Despite being mitotically quiescent (16, 17), the dramatic rise in FLc numbers is considered to be contributed by the constant differentiation of multiple stem/progenitor cells (18). Several factors like NR5A1 or SF1, dosage-sensitive sex reversal, adrenal hypoplasia critical region, on chromosome X, gene 1 (DAX1), desert hedgehog (DHH), platelet-derived growth factor- A (PDGF-A), orphan nuclear receptor chicken ovalbumin upstream promoter transcription factor II (COUP-TFII), insulin-like growth factor 1 (IGF-1), hepatocyte growth factor (HGF), homeobox gene Aristaless (ARX), etc. regulate this differentiation process (19). Double mutants of *Sf-1* and *Daz1* show a complete loss of FLc suggesting a synergistic effect of these two factors on FLc differentiation (20). Both DHH and PDGF-A are derived from fetal Sc and act *via* respective receptors e.g.- Patched *(Ptch) 1* and PDGF-α expressed by interstitial stem/progenitor cells (18, 19). Furthermore, data from either *Dhh*-null mouse (showing reduced numbers of FLc) (21) or over-expressing/constitutively activated DHH-induced downstream signaling molecule *Gli 1* (Gli- Kru¨ppel family of transcription factor1) (22) and Smo (23) (showing hyperplasia of FLc) confirmed the crucial inductive role of DHH signaling in FLc differentiation. COUP-TFII plays a critical role in maintaining the FLc pool either by promoting the differentiation of FLc-progenitor cells (24) or by repressing the functional maturation of FLc *via* antagonizing SF1 (25). However, constitutive activation of Notch signaling in fetal testes leads to a dramatic decrease in FLc number, suggesting Notch signaling (downstream target *Hes1*) being a negative regulator of FLc differentiation (26). Notably, vasculature-dependent Notch signaling has been shown to regulate the critical balance between self-renewal and differentiation of FLc stem/progenitor cells (13). Firstly, FLc-progenitors that are derived from coelomic epithelium progressively lose Notch activation as they ingress into fetal testes, thereby directly get differentiated to FLc (13). Secondly, FLc-progenitors that originate from mesenchymal-perivascular progenitors get exposed to high Notch signal [since they co-migrate with JAG-1-positive (Notch-ligand) endothelial cells from the mesonephros into nascent testis] thereby serving as a potential pool of Lc-stem cells *via* self-renewal (13).

### 2.3 Key features & functions

Differentiated FLc are found to be transcriptionally robust as compared to that of the non-steroidogenic, interstitial, SF1-positive stem/progenitor cells. To be specific, the transcription of essential enzymes/cofactors involved in energy-generating critical metabolic pathways e.g. – glycolysis, TCA cycle, oxidative phosphorylation etc. are found to be augmented in FLc (27). Despite being responsive towards embryonic pituitary LH, functions of FLc are completely independent of gonadotropins (10). Notably, unlike ALc, mouse FLc express 11β-hydroxylase (*Cyp11β1*) & 21-hydroxylase (*Cyp21*) however lack 17β-hydroxysteroid dehydrogenase type 3 (*Hsd17β3*). Therefore, although they can respond to ACTH signals but fail to convert androstenedione to T (10). In fetal mouse testes, androstenedione gets converted totestosterone by Sc (10). In mice, intra-testicular T is first detectable at E 13.0 days then peaks at E 17-19 days prior or during the time of birth (1). *In utero* life,testosterone induces the initial virilization of the male external genitalia (1). The other critical hormone produced by FLc is INSL3, a member of the insulin-relaxin family of peptides which operates *via* G-protein coupled receptor relaxin/insulin-like family peptide receptor 2 (RXFP2). Null mutations in either *Insl3* or *Rxfp2* result in cryptorchidism (28, 29). On the other hand, transabdominally descended ovaries are found in female mice over-expressing *Insl3* (30, 31).

### 2.4 Fate in post-natal testes

The differentiation of FLc occurs throughout the fetal life, peaking during birth, gradually declining by the first two weeks of postnatal life, and subsequently disappears completely during pre-pubertal age (1). The concentration of intra-testicular (and in systemic circulation)T also drops along with the progressive regression of FLc in neonatal mouse (32). However, external supplementations of T from day 7 to 11 of postnatal age substantially augments Notch signalling in Androgen Receptor (AR) expressing perivascular cells indicating that T may provide a feedback response to maintain the FLc stem/progenitor pool (32). Classical histological studies (33–35) and recent lineage tracing experiments (36) have demonstrated that a sub-fraction of FLc are retained in adult testes, contributing around 5–20% of the total Lc population. This unique population of FLc persistent in adult testes remains functionally debatable as they remain unresponsive towards T, despite sharing comparable transcriptomic profiles with that of ALc (37, 38).

## 3 ALc

### 3.1 Origin, differentiation and capacity of regeneration

Since it has been claimed that FLc are replaced by ALc in pre-pubertal mouse testes, researchers continue to argue on the presence of a common stem/progenitor pool for both FLc and ALc (18, 19) or alternatively having a unique stem/progenitor system specific to ALc (39). Although FLc number remains unaffected in AR knockout (AR-KO) mice, no ALc is detectable in adult testes indicating unlike, FLc, T plays a crucial rolein ALc differentiation (10). Furthermore, fetal androgen deficiency leads to compromised ALc function during adulthood indicating a critical role of T in programming these interstitial stem/progenitor cells during fetal life (40). As discussed in the earlier section, these stem/progenitor cells are non-steroidogenic, interstitial cells of multiple lineages e.g.- SF1-positive cells (18, 19) or Nestin-positive perivascular cells (13) having mesenchymal-like morphological appearances. Like FLc, DHH, PDGF-A, and COUP-TFII are considered to be the critical regulators of ALc differentiation (10, 18, 19). *Dhh* ablation with *Sf1* haploinsufficiency leads to the complete loss of both FLc and ALc (41). Furthermore, insulin-like growth factor I (IGF1), Leukemia inhibiting factor (LIF), and c-kit-ligand/Stem cell Factor (SCF) are also reported to be essential for the functional maturation of ALc (10). The presence of a potent stem/progenitor pool for ALc has been confirmed by the regeneration of ALc within 3 months of administration of alkylating agent ethylene sulfonate (EDS) in adult rat testes (42–45). It is evident that EDS can selectively ablate ALc only and the Lc-stem/progenitor cells remain insensitive towards it (44, 45). However, external administration of T inhibits/delays such regenerative process confirming T acts on perivascular cells and suppresses the differentiation of Lc-stem/progenitor cells (13, 32). Some studies also claim that the peritubular-myoid cells may serve as the precursor of ALc, too (18). Sc-derived Anti Mullerian Hormone (AMH) has been shown as a negative regulator of Lc-stem/progenitor cells (10). AMH over-expressing (46) or AMH-KO (47) adult mice have been shown to have poor ALc number and Lc hyperplasia respectively. Characterization of these stem/progenitor cells of ALc has also been achievable by isolation and culture from 7-days-old neonatal rat testes. These cells continue to show indefinite proliferative capacity (via self-renewal) in long-term culture, thyroid hormone, IGF1, and LH, stimulated *in vitro* maturation with elevated expression of 3β-HSD & production of T and post-transplantation colonizing ability to the host-testicular interstitium and subsequent differentiation to ALc *in vivo* (48).

### 3.2 Uniqueness

As compared to FLc-morphology, ALc have relatively lower lipid droplets with a robust network of smooth endoplasmic reticulum (sER) and tubule-vesicular mitochondria, etc. (10). Moreover, unlike FLc, ALc are ACTH insensitive and the development and function of ALc are completely dependent on LH and T (49). Recent studies have demonstrated the requirement of AR in LH-induced differentiation of ALc by inhibiting the adrenal characteristics in the testicular interstitium (50). The expressions of StAR, Cyp11a1, Cyp17a1, 3β-HSD type 6, and 17β-HSD type 3 are substantially higher in ALc as compared to FLc (1). Finally, unlike FLc, the maturing/differentiating ALc are mitotically active and TGFβ, IGF1, NGF, etc signaling pathways promote such proliferation (10).

### 3.3 Function

In the adult male, androgens critically maintain masculinity (sex drive/erectile function) and fertility (1). ALc expresses both LH-receptor (LH-R) and AR (10). LH critically regulates Lc steroidogenesis (biosynthesis of testicular androgen, T) and T acts on ALc *via* an autocrine fashion (1). Although Lc-specific AR knockout (KO) mice are found to be sub-fertile (51, 52), both LH-β (53) and LH-R (54) null mice are sterile, indicating critical endocrine dependence of ALc.T is absolutely required for pubertal maturation of Sc (55), the establishment of blood-testis barrier (BTB) (56), progression and completion Gc meiosis and spermiation (57).

## 4 Difference between rodent and primate Lc

Rodent and primate Lc differ in structure, development and function (10). There are three fundamental differences observed in human FLc as compared to that of the rodents.

Firstly, FLc of both the species are independent of fetal LH action, despite being responsive towards LH signal (58). Mouse FLc express LH-R by E 16 days (10), whereas in humans LHCG-R (both for LH and hCG) is detectable in testes by the 11th week of gestation (10). FLc number or external genitalia are unaffected in hpg (hypogonadal lacking GnRH) (59), LH-R (54), LH-β (53) and ARKO (60, 61) adult male mice suggesting murine FLc are functionally independent of LH or T. In humans, T concentration peaks during 12-14 weeks of gestation fetal circulation coinciding with placental hCG which is around 10-fold higher than pituitary LH (10). The decline in fetal T is also well-correlated with the drop in circulatory hCG during the second trimester (10). Furthermore, although patients having LH-β mutations show normal masculinized development (62, 63), LHCG-R mutations lead to pseudo-hermaphroditism (64) indicating a definite role of hCG on FLc functioning in men.

Secondly,T is the major androgen in fetal murine testes (FLc produces androstenedione *via* the canonical pathway which gets converted toT in Sc).However, dihydrotestosterone (5α-DHT) is a more potent/bioactive androgen recently reported to be critical for the virilization of human male external genitalia (1). In the reproductive tract of adult men,T gets bio-converted to 5α-DHT by 5α-reductase type 2 (coded by the *Srd5a2* gene) and men with inactive *Srd5a2* mutations have ambiguous genitalia (10). However, recent studies have indicated that an alternative backdoor pathway is operational in human male fetal testes where 5α-DHT is biosynthesized [without getting converted to dehydroepiandrosterone (DHEA), using 5α-dihydro-progesterone, allopregnanolone,17-hydroxyallopregnanolone, androsterone and androstanediol as intermediates] from androstanediol by alfa-keto-reductases (coded by *Akrc2* and *Akrc4*) (65). The critical role of this DHEA-independent backdoor pathway has been established from comparable birth defects found in men with *Srd5a2* or *Akrc2*/*4* inactivating mutations (65, 66). Intriguingly, male fetal genital development and fertility remain unaffected in *Hsd17β3*null mice having complete ablation of canonical production ofT (via intermediates like DHEA and androstenedione) (67).

Finally, unlike mice and rats, primate Lc development is triphasic (68, 69). In humans, FLc gets dedifferentiated by the end of the second trimester with a decline of hCG, very few FLc successfully escape this event and remain active during the time of birth (69). A unique population of neonatal-Lc (NLc) is reported in neonatal/infant boys for the first 4-6 months of age when the hypothalamic-hypophyseal testicular (HHT) axis remains active (70). These NLc are morphologically comparable to FLc with anastomosing sER, pleomorphic mitochondria, extensive trans-Golgi network, etc. (69). However, like ALc these NLc lack cytoplasmic reticulum Reinke crystals (69).Multiple claims have been postulated on the fate of NLc, e.g. during the onset of the juvenile period (inactive HHT axis) massive involution occurs in NLC population, NLc undergoes a partial regression at the end of in the infantile period and a limited fraction of NLc survive synthesizing a low basal T throughput the juvenile age (69). The ALc population originates from the dedifferentiating NLc population or directly differentiates from the stem/progenitor cells (69).

## 5 Lc aging

Progressive decline in T production is manifested with testicular aging (71–74). Studies in both humans and rats have demonstrated that the efficacy of LH-induced T production gets diminished with testicular age, despite no significant change in the number of ALc (75, 76). Features of Lc aging include morphological alteration of sER and mitochondrial structure, compromised LH-R signaling/responsiveness, impaired cholesterol trafficking, poor expression and activity of steroidogenic enzymes, accumulation of reactive oxygen species (ROS) due to metabolic impairment/disruption of the pro/antioxidant balance and deficiency in autophagy leading to poor cellular homeostasis (75). Furthermore, the EDS–induced regenerative capacity of the Lc-stem/progenitor population is also not maintained for the long term indicating a probable influence of the aged microenvironment on Lc function (75). A most recent model of premature aging having constitutive CISD2 (CDGSH iron sulfur domain 2, a redox-active protein of ER) deficiency confirmed that the age-related dysfunction of Lc is not completely intrinsic but largely depends on the associated supportive microenvironment of the testes (77).

## 6 Conclusion and future directions

**Figure 1:**
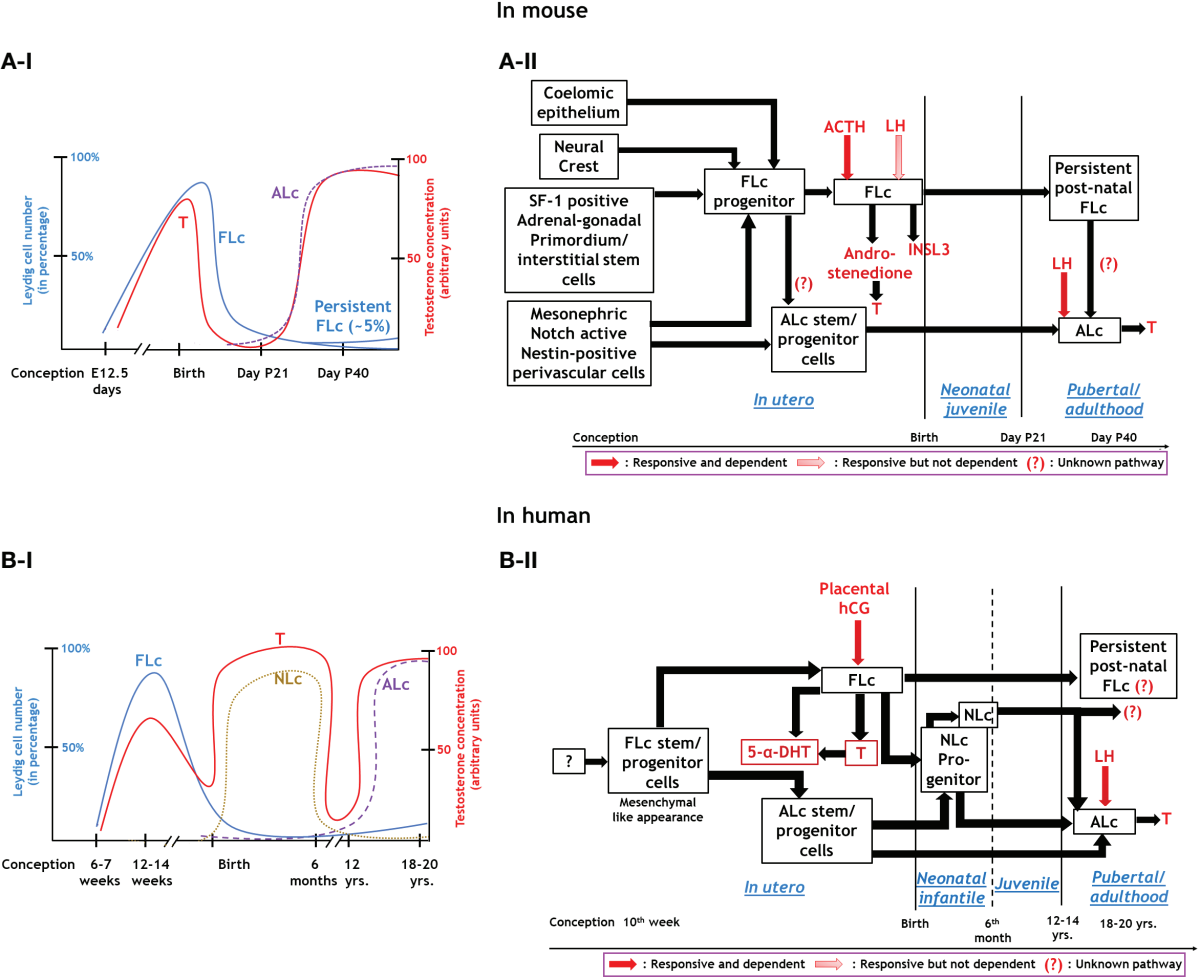
Endocrinal regulation and functions of three major types of Leydig cells. Panel A: In mouse; A-I: Relationship between the development of FLc and ALc with blood testosterone level during various phases of murine life. A-II: Synthesis, hormonal regulation, and function of Leydig cells in mice. Panel B: Human; B-I: Association between FLc, NLc, and ALc development and blood testosterone levels throughout human life. B-II: Synthesis, hormonal regulation, and function of different lineages of Leydig cells in humans.

In summary, both traditional histological studies and modern high-throughput multi-omics approaches using genetically manipulated mice models have revealed the diverse origin of Lc during testicular development (8–10, 12, 13). Both FLc and ALc may be lineage independent of each other or share multiple common cellular precursor cells (18, 19). However, both of them significantly differ in terms of structure and function (1). The migration of mesonephric endothelial cells to the nascent testis establishes a testis-specific vasculature pattern which is considered to be the prerequisite of testicular cord formation- precursor structure of seminiferous tubules (11, 78). This testicular vascular niche recently has been shown to direct the Notch signaling to maintain the critical balance between perivascular- stem/progenitor cells and differentiating Lc (12, 13). The existence of persistently present androgen-insensitive FLc in adult testes also has been established (36). Although FLc from rodents or primates is independent of fetal LH signaling, human FLc is fully dependent on placental hCG (10). However, both LH andT signaling are critical regulators of ALc function in both species (10). **Figure 1** schematically represents the developmental schedule of Lc in both mouse (A-I, A-II) and human (B-I, B-II) discussed here, whereas **Table 1** summarizes the critical difference between FLc and ALc in both mouse (A) and human (B).

**Table 1:** Cross-species comparative study on different Leydig cell functions.

1A: Comparison between properties and functions of murine fetal and adult Leydig cells.		
Key points	Fetal Leydig cell (FLc)	Adult Leydig cell (ALc)
*Developmental Time of Occurrence*	Appear during Embryonic age (E) 13.5-15.5 days, peak at E 17-19 days, remain up to Post-natal age (P) 15-21 days, a small fraction (5-20%) persistent in adult testes	Appear during P 15-21 days, peak by P 30-40 days, remain constant throughout adulthood
*Origin*	Coelomic epithelium, neural crest, notch active, Nestin-positive perivascular cells located at the gonad-mesonephros border region, interstitial Nr5a1or Ad4BP/SF1-positive and WT1 - positive stem/progenitor cells	FLc, notch active, Nestin-positive interstitial perivascular cells, interstitial Nr5a1or Ad4BP/SF1-positive cells
*Inducer*	SF1, DAX1, DHH, PDGF-A, COUP-TFII, IGF-1, HGF, ARX, etc.	SF1, DHH, PDGF-A, COUP-TFII, IGF-1, LIF, SCF, Thyroid hormone, etc.
*Repressor*	Notch, COUP-TFII	Notch, AMH, EDS, etc.
*Morphology*	High lipid droplets	Lower lipid droplets with a robust network of smooth endoplasmic reticulum (sER) and tubule-vesicular mitochondria
*Mitotic activity*	Not Active	Differentiating ALc are active by IGF-1, TGF-β, NGF, etc., and differentiated ALc are inactive.
*ACTH responsiveness*	Responsive	Insensitive
*LH dependence*	Responsive but independent	Completely dependent
*T dependence*	Independent	Significantly dependent
*Regenerative capacity*	Not Established	Well established, within 3 months of EDS exposure
*Steroidogenic markers*	Like the adrenal cortex, high *Cyp11β1*&*Cyp21*but no 17β-HSD type 3, etc.	High StAR, Cyp11a1, Cyp17a1, 3β-HSD type 6, 17β-HSD type 3 etc.
*Major hormones secreted with functions*	Androstenedione (gets converted to T by fetal Sc) for initial virilization of the male external genitalia and INSL3 for testicular descent.	T (which gets converted to 5α-DHT in the male genital tract) for maintaining masculinity and fertility (pubertal maturation of Sc etc., meiotic progression of Gc, spermiation, sex drive/libido, etc.).

**Table:** 

1B: Comparison amongfunctions of mouse and human FLc, NLc and ALc.		
Key points	Mouse	Human
*Mode of Development*	Biphasic	Triphasic
FLc	i) Though responsive but independent of fetal LH and/or ii) Synthesize Androstenedione iii) Persistent in adult testes	i) Though responsive but independent of fetal LH, but completely dependent on placental hCG ii) Synthesize T and 5α-DHT (via DHEA independent backdoor pathway) iii) Completely absent in pubertal/adult testes
NLc	No such transitional stage reported	During neonatal/infantile life with a robust HHT axis for the first 4-6 months of post-natal age
ALc	Originate from either FLc or notch active, Nestin-positive interstitial perivascular cells, interstitial Nr5a1or Ad4BP/SF1-positive cells	Originate from either NLc or interstitial stem/progenitor cells

Recent advancements in targeted reprogramming [like selective ablation of Wt1 in Ctnnb1 (cadherin-associated protein β1) over-expressing Sc results into Lc cell-like tumor development (79), manipulation of SF1, GATA4,etc. in either fibroblast cells (80) or in induced human pluripotent stem cells (81) leads to T producing Lc- like cells,etc.] has revolutionized the field with a potential clinical promise for cell-based therapy for hypogonadism (82, 83). However, more in-depth studies are still required in different stages of testicular maturation (fetal, neonatal, pre-pubertal, and adult) using lineage tracing and single-cell RNA-seq approaches to reveal unique molecular markers for each different stage of Lc differentiation of each lineage including Lc-stem/progenitor cells. Testicular macrophages [critical for fetal testicular morphogenesis (84) and spermatogonial differentiation (85)] are developmentally coupled with interstitial Lc (86, 87). Sincere efforts are also to be made in the future to investigate the molecular crosstalk between these two cells with respect to testicular development and aging.

## Author contributions

IB conceived the idea and designed the initial draft. IB prepared the text and figure and generated the final form with active support from SD. SD revised the manuscript. All authors contributed to the article and approved the submitted version.
